# Associations of preoperative stroke and tranexamic acid administration with convulsive seizures in valvular open-heart surgery

**DOI:** 10.1007/s00540-021-02924-w

**Published:** 2021-04-06

**Authors:** Nikolai Hulde, Armin Zittermann, Marcus-André Deutsch, Vera von Dossow, Jan F. Gummert, Andreas Koster

**Affiliations:** 1 Institute of Anesthesiology and Pain Therapy, Herz- und Diabeteszentrum NRW, Ruhr-University Bochum, Bad Oeynhausen, Germany; 2 Clinic for Thoracic and Cardiovascular Surgery, Herz- Und Diabeteszentrum NRW, Bad Oeynhausen, Ruhr-University Bochum, Georgstr. 11, 32545 Bad Oeynhausen, Germany

**Keywords:** Tranexamic acid, Seizure, Stroke, In-hospital mortality, 30-day mortality

## Abstract

In cardiac surgery, use of the antifibrinolytic agent tranexamic acid (TXA) and acute perioperative stroke are both associated with convulsive seizures. We hypothesized that an older (preoperative) stroke increases the risk of TXA-associated seizures as well. To test this hypothesis, we retrospectively analyzed data from 16,110 patients who had undergone open-heart valvular surgery at our institution between 2009 and 2020. The dosing of TXA was moderate. Use of TXA and a history of stroke were both independently associated with convulsive seizure with an adjusted odds ratio (OR) of 2.40 (95%CI: 1.71–3.37) and 1.79 (95%CI: 1.27–2.54), respectively. Compared to patients without TXA administration, the adjusted OR of experiencing a seizure in TXA patients without a history of stroke was 2.44 (95%CI: 1.71–3.46) and in patients receiving TXA with a history of stroke 4.30 (95%CI: 2.65–6.99). However, there was no significant interaction between TXA use and preoperative stroke on convulsive seizures (*P* = 0.77). Compared to patients without seizure, for patients with seizure, the inverse probability-weighted ORs of in-hospital mortality and 30-day mortality were 3.58 (95%CI: 2.20–5.83) and 4.04 (95%CI: 2.34–6.98), respectively. We conclude that, in patients undergoing open-heart surgery, a history of stroke is independently associated with convulsive seizures but is not a contraindication for TXA use.

**Supplementary Information:**

The online version contains supplementary material available at 10.1007/s00540-021-02924-w.

## Introduction

In cardiac surgery, the antifibrinolytic agent tranexamic acid (TXA) has been associated with convulsive seizures [1]. The underlying pharmacologic mechanism is considered to be a disinhibition of cerebral glycine and gamma-aminobutyric acid receptors [2]. To date, increasing TXA dose and open-heart surgery are the only parameters that have been clearly established as underlying risk factors of convulsive seizures [1–3]. In the large randomized, placebo-controlled ATACAS (Aspirin and Tranexamic Acid for Coronary Artery Surgery) trial that included patients undergoing isolated valvular or combined valvular and coronary artery bypass grafting (CABG) surgery, use of TXA (100 mg and 50 mg per kg body weight in 75% and 25% of trial participants, respectively) was associated with a higher risk of postoperative seizures than in the placebo group [3]. In addition, a strong positive relationship between a perioperative stroke and a seizure was observed [3]. We therefore hypothesized that a history of stroke may also be a critical risk factor for TXA-associated seizures. To test this hypothesis, we analyzed data from our high-volume center over the last 10 years.

## Methods

### Patient selection and anesthesia technique

We performed a retrospective analysis in patients who had undergone either isolated valvular or combined valvular and CABG open-heart surgery at our institution between July 2009 and September 2020. Patients with concomitant aortic surgery were excluded. After further exclusion of 47 patients with missing pre- or perioperative data, a total of 16,110 patients could ultimately be analyzed (Table 1). Anesthesia was introduced with etomidate, rocuronium and sufentanil and maintained with a continuous infusion of remifentanil (0.5–1 µg/kg/min) and vaporization of sevoflurane. After closure of the chest, sevoflurane was replaced by a continuous infusion of propofol (2–4 mg/kg/h). The decision to give TXA was agreed between the surgeon and the anesthetist based on the degree of microvascular bleeding before heparinization. TXA was given with a bolus of 1 g after heparinization, followed by an infusion of 0.2 g/h until the end of cardiopulmonary bypass. In the priming volume of the cardiopulmonary bypass system, 0.5 g of TXA was added. For antibiotic prophylaxis, 2 g of cefazolin was given after the induction of anesthesia, and thereafter at intervals of 3 h.

**Table 1:** Baseline characteristics by subgroups of preoperative stroke and perioperative tranexamic acid administration

Parameter	Non-stroke, non-TXA *n* = 3136	Stroke, non-TXA *n* = 95	Non-stroke, TXA *n* = 12,305	Stroke, TXA*n* = 574	*P* value
Age (years)	71.4 (61.3;77.2)	72.9 (63.0;78.4)	71.4 (61.9;77.3)	72.6 (63.6;77.6)	0.13
Sex, Males	1812 (57.8)	57 (60.0)	7666 (62.3)	363 (63.2)	< 0.001
Body mass Index (kg/m^2^)	26.3 (23.7;29.4)	26.6 (23.4;30.1)	26.8 (24.1;30.1)	26.9 (24.2;30.5)	< 0.001
Diabetes mellitus	580 (18.5)	16 (27.4)	2770 (22.5)	186 (32.4)	< 0.001
Hypertension	2261 (72.1)	79 (83.2)	9625 (78.2)	467 (81.4)	< 0.001
Myocardial infarction	196 (6.3)	13 (13.7)	1082 (8.8)	84 (14.6)	< 0.001
Chronic obstructive pulmonary disease	243 (7.7)	8 (8.4)	1166 (9.5)	61 (10.6)	0.015
Left ventricular ejection fraction (%)	60 (55;65)	55 (50;60)	60 (50;64)	55 (50;60)	< 0.001
Estimated glomerular filtration rate (ml/min/1.73m^2^)	70.8 (54.5;83.9)	64.2 (44.2;81.4)	68.8 (53.4;82.2)	63.9 (43.9;79.7)	< 0.001
Hemoglobin (g/l)	13.7 (12.5;14.7)	12.9 (11.3;14.0)	13.6 (12.2;14.7)	12.5 (10.8;13.9)	< 0.001
Peripheral Artery Disease	136 (4.3)	11 (11.6)	749 (6.1)	73 (12.7)	< 0.001
Three vessel disease	342 (10.9)	20 (21.1)	2,404 (19.5)	139 (24.2)	< 0.001
Carotid stenosis > 50%	122 (3.9)	9 (9.5)	703 (5.7)	64 (11.1)	< 0.001
Euroscore II (%)	1.8 (1.0;3.6)	4.6 (2.1;13.3)	2.4 (1.2;5.1)	5.7 (2.5;14.9)	< 0.001
Aspirin use	743 (23.7)	28 (29.5)	4,519 (36.7)	252 (43.9)	< 0.001
Smoker/previous smoker	859 (27.4)	28 (29.5)	3,491 (28.4)	153 (26.7)	0.59
Previous cardiac surgery	144 (4.6)	15 (15.8)	1,642 (13.3)	138 (24.0)	< 0.001
Operation priority					< 0.001
Elective	2953 (94.2)	67 (70.5)	11,242 (91.4)	395 (68.8)	
Urgent	91 (2.9)	10 (10.5)	572 (4.6)	63 (11.0)	
Emergent	91 (2.9)	18 (18.9)	490 (4.0)	116 (20.2)	
Isolated valve surgery	2478 (79.0)	73 (76.8)	8457 (68.7)	373 (65.0)	< 0.001
Combined valve plus CABG surgery	658 (21.0)	22 (23.2)	3,848 (31.3)	201 (35.0)	< 0.001

Data are presented as median with 25th and 75th percentiles or number with percent

### Data collection and study design

We assessed 19 preoperative patient characteristics (Table 1) and three outcome parameters (seizure, in-hospital mortality, 30-day mortality), in addition to the two parameters history of stroke and use of TXA. A seizure was considered to have occurred in the case of sudden clonic movement of the patient and was verified by a computer tomography scan. For data analysis, the study cohort was divided into four subgroups: patients with or without preoperative stroke and with or without perioperative TXA administration.

### Statistical analysis

Categorical variables are summarized as percentages and number of observations. Preoperative continuous variables are presented as median with 25th and 75th percentiles. We used the Mann–Whitney *U* test and Fisher’s exact test to assess group differences in continuous and categorical variables where appropriate. We performed univariate and multivariable-adjusted binary logistic regression analysis to assess the association of study group with perioperative seizure risk (see Supplemental Material). Data are presented as odds ratio (OR) with 95% confidence interval (CI). A sample size calculation with Bonferroni correction for multiplicity revealed that with 500 patients in the non-stroke, non-TXA group and 500 patients in the stroke, TXA group, there will be a 90% chance of detecting a significant difference at a two-sided 0.0125 significance level. This assumes incidence rates of 1% and 5%, respectively. We also tested for interaction (based on the Wald test for interaction term) between TXA administration and preoperative stroke with adjustment of covariates. To assess associations of seizures with in-hospital and 30-day mortality, we applied the method of inverse probability weighting (see Supplemental Material). *P* values < 0.05 were considered statistically significant. We used the statistical software package IBM SPSS, version 27 (IBM Corp, Armonk, NY, USA), to perform the analyses.

## Results

Use of TXA and a history of stroke were both independently associated with convulsive seizure (Supplemental Table 1). The four groups of patients with or without preoperative stroke and with or without perioperative TXA administration differed substantially with respect to most baseline characteristics assessed, with the exception of age and smoking status (Table 1). Compared to patients without TXA administration, the adjusted OR of experiencing a seizure in TXA patients without a history of stroke was 2.44 (95%CI: 1.71–3.46) and in patients receiving TXA with a history of stroke 4.30 (95%CI: 2.65–6.99) (Table 2). There was, however, no significant interaction between TXA use and preoperative stroke on convulsive seizures (*P* = 0.77).

**Table 2:** Odds ratios of perioperative seizures by subgroups of preoperative stroke and perioperative tranexamic acid administration

Parameter	*n*	Seizure *n* (%)	Unadjusted ORs (95%CI)	Multivariable-adjusted ORs^1^ (95%CI)
Subgroup				
Non-stroke, Non-TXA	3,136	35 (1.1)	1.0 (reference)	1.0 (reference)
Stroke, non-TXA	95	3 (3.2)	2.89 (0.87–9.57)	2.13 (0.63–7.11)
Non-stroke, TXA	12,305	358 (2.9)	2.66 (1.87–3.77)	2.44 (1.71–3.46)
Stroke, TXA	574	36 (6.3)	5.93 (3.69–9.53)	4.30 (2.65–6.99)

*TXA*tranexamic acid; *ORs* odds ratios; *CI* confidence interval

^1^Adjustments were made for age, body mass index, left ventricular ejection fraction, estimated glomerular filtration rate, hemoglobin, carotid stenosis, and previous cardiac surgery

In-hospital mortality and 30-day mortality were 3.4% (*n* = 547) and 3.5% (*n* = 569), respectively. Compared with patients without seizure, for patients with seizure, inverse probability-weighted in-hospital mortality and 30-day mortality were 3.58 (95%CI: 2.20–5.83) and 4.04 (95%CI: 2.34–6.98), respectively.

## Discussion

Our results indicate that a history of stroke and use of TXA are both independently associated with an increased risk of seizure in patients undergoing valvular open-heart surgery. However, our data do not support the hypothesis of a significant interaction of both parameters on the risk of seizure.

It is well known that, in open-heart surgery, TXA dose-dependently increases the risk of convulsive seizure [1, 3]. This is attributed to toxic TXA concentrations (≥ 200 µM) in the cerebral fluid. The underlying cause is considered to be an increased permeability of the blood–brain barrier due to systemic inflammation and its jeopardization via micro-air embolism following open-heart surgery or cerebral injury [2]. With respect to acute stroke, dysfunction of the blood–brain barrier is a prominent characteristic, and it is unclear whether complete restoration occurs [4]. Therefore, a history of stroke may increase the risk of seizure by persistent blood–brain barrier impairment, particularly in the aged brain [4]. However, the concept of an impaired blood–brain barrier has been challenged by the results of two recent large randomized placebo-controlled trials in acute traumatic brain injury and acute intracerebral hemorrhage [5, 6]. In both trials, the dosing of TXA was almost comparable to our protocol. The CRASH-3 (Clinical Randomisation of an Antifibrinolytic in Significant Haemorrhage) trial allocated 12,737 patients with traumatic brain injury to TXA or matching placebo [5]. The risk of developing seizures did not differ significantly between groups [5]. The TICH-2 (Tranexamic acid for hyperacute primary IntraCerebral Haemorrhage) trial allocated 2325 patients with acute spontaneous intracerebral hemorrhage to TXA or matching placebo [6]. The incidence of seizures in both groups was 7% [6]. These data show that the mechanisms leading to toxic TXA concentrations in cardiac surgery and potentially promoting stroke-related convulsive seizures require further research. The results of the two aforementioned studies are also in line with our finding that there was no significant interaction between a history of stroke and TXA administration on seizures. Obviously, there is no similar mechanism of how both parameters may influence the risk of seizures.

It should be emphasized that our results of a higher short-term mortality risk in patients with seizures than in patients without seizures are in line with earlier findings [3].

Our investigation has several limitations that have to be addressed. First, the retrospective study design prevents us from assuming causal relationships between risk factors and outcomes. Second, the small numbers of patients in the two groups with a history of stroke may have reduced statistical power for detecting significant interactions between use of TXA and a history of stroke on convulsive seizures. Third, we cannot rule out that study results are biased by unexplained confounding. Finally, the propensity score for calculating inverse probability weighting was solely based on preoperative covariate data and did not include perioperative parameters.

In summary, our data indicate that, in patients undergoing open-heart surgery, a history of stroke is independently associated with convulsive seizures. Since there was no significant interaction between TXA use and preoperative stroke on convulsive seizures, a history of stroke is not a contraindication for TXA use.

## Supplementary Information

Below is the link to the electronic supplementary material.

Supplementary file1 (PDF 94 KB)
