# Correction to: Associations of preoperative stroke and tranexamic acid administration with convulsive seizures in valvular open-heart surgery

**DOI:** 10.1007/s00540-021-02936-6

**Published:** 2021-04-28

**Authors:** Nikolai Hulde, Armin Zittermann, Marcus-André Deutsch, Vera von Dossow, Jan F. Gummert, Andreas Koster

**Affiliations:** 1 Institute of Anesthesiology and Pain Therapy, Herz- und Diabeteszentrum NRW, Ruhr-University Bochum, Bad Oeynhausen, Germany; 2 Clinic for Thoracic and Cardiovascular Surgery, Herz- Und Diabeteszentrum NRW, Bad Oeynhausen, Ruhr-University Bochum, Georgstr. 11, 32545 Bad Oeynhausen, Germany

## Correction to: Journal of Anesthesia 10.1007/s00540-021-02924-w

In the original publication of the article, corresponding author’s last name was published incorrectly as “Zitttermann”. The correct name is “Armin Zittermann”.

The original article has been corrected.

