# Supplementary material for: Associations of preoperative stroke and tranexamic acid administration with convulsive seizures in valvular open-heart surgery
Source: J Anesth. 2021 Apr 6;35(3):451–4. doi: 10.1007/s00540-021-02924-w (PMC8124038; doi:10.1007/s00540-021-02924-w)
Supplement: Supplementary file 1 — Supplementary file1 (PDF 94 KB) [file 540_2021_2924_MOESM1_ESM.pdf]

Associations of Preoperative Stroke and Tranexamic Acid Administration with Convulsive Seizures in Valvular Open-Heart Surgery

Journal of Anesthesia

Nikolai Hulde, Armin Zitttermann[1], Marcus-André Deutsch, Vera von Dossow,

Jan F. Gummert, and Andreas Koster

[1]Corresponding Author:
Armin Zittermann, PhD
Clinic for Cardiothoracic Surgery
Herz- und Diabeteszentrum NRW
Ruhr University Bochum
D-32545 Bad Oeynhausen
Georgstr. 11
E-mail: azittermann@hdz-nrw.de
Phone: +49 5731 97-3296
Fax: +49 5731 97-2020

Statistical Analysis

The association of preoperative factors with convulsive seizures was first tested in a univariate logistic regression model. Every univariate variable showing significant ($P < 0.05$) association with the risk of seizure was then tested in a multivariable logistic regression model and removed stepwise if no significant influence was proved. The following 21 parameters were tested: age, sex, body mass index, diabetes mellitus, hypertension, myocardial infarction, chronic obstructive pulmonary disease, left ventricular ejection fraction, estimated glomerular filtration rate, hemoglobin, peripheral artery disease, three vessel disease, carotid stenosis, Euroscore II, aspirin use, smoking status, previous cardiac surgery, operation priority, type of surgery, history of stroke, and use of tranexamic acid. Only those variables which were significantly ($P<0.05$) associated with clinical outcome remained in the multivariable model. The Hosmer-Lemeshow test was used to test for goodness of fit for the logistic regression models. The risk factor analysis of convulsive seizures is presented in Supplemental Table 1. The model was sufficiently calibrated among deciles of observed and expected risk (Hosmer-Lemeshow test $p = 0.37$).

The association of seizures with short-term mortality was assessed using inverse probability weighting. The following formula was applied: $T/PS + (1 - T)/(1 - PS)$, whereas T indicates patient's status being 0 in patients without seizure and 1 in patients with seizure. PS indicates the propensity score of each patient. The propensity score was based on the aforementioned 21 preoperative parameters.

Supplemental Table 1: Risk factor analysis of convulsive seizure in patients undergoing valvular open-heart surgery

Parameter	Univariate OR (95%CI)	Multivariable OR (95%CI)	P-value[1]
Age (per year)	1.04 (1.03-1.05)	1.03 (1.02-1.04)	<0.001
Female Sex	1.41 (1.16-1.71)	-	
Body Mass Index (per unit)	0.95 (0.93-0.98)	0.95 (.93-0.97)	<0.001
Three-Vessel Disease (reference: no)	1.15 (1.07-1.24)	-	
Peripheral Artery Disease (reference: no)	1.86 (1.35-2.55)	-	
Carotid Stenosis (reference: no)	1.92 (1.39-2.64)	1.42 (1.02-1.98)	0.037
Hypertension (reference: no)	1.35 (1.05-1.73)	-	
Stroke (reference: no)	2.35 (1.68-3.30)	1.79 (1.27-2.54)	0.001
Previous Cardiac Surgery (reference: no)	1.85 (1.57-2.19)	1.45 (1.22-1.73)	<0.001
Left Ventricular Ejection Fraction (per %)	0.98 (0.97-0.99)	0.99 (.98-1.00)	0.009
Estimated Glomerualr Filtration Rate (per ml/min/1.73 m^2)	0.98 (0.97-0.98)	0.98 (0.98-0.99)	<0.001
Hemoglobin (per g/dl)	0.82 (0.79-0.86)	0.92 (0.88-0.97)	0.02
Euroscore II (per unit)	1.03 (1.03-1.04)	-	
Operation Priority		-	
Elective	1.0 (reference)		
Urgent	1.96 (1.38-2.80)		
Emergent/Ultima ratio	1.90 (1.32-2.74)		
Use of Tranexamic Acid	2.65 (1.89-3.70)	2.40 (1.71-3.37)	<0.001

[1]only significant results in multivariable-adjusted analysis are presented. No significant association with convulsive seizure in univariate analysis was present for: diabetes mellitus, chronic obstructive pulmonary disease, myocardial infarction, smoking status, aspirin use, and surgical group, Abbreviations: OR, odds ratio, CI, confidence interval
